# Single-access percutaneous coronary intervention with IMPELLA CP support using a 16F sheath in refractory ventricular fibrillation: a case report

**DOI:** 10.1186/s43044-026-00717-8

**Published:** 2026-02-02

**Authors:** Yuki Sunami, Takumi Toya, Takafumi Nishimura, Masayuki Aoyama, Yoshichika Miyazaki, Munehisa Sakamoto

**Affiliations:** 1 Division of Cardiology, NHO Tokyo Medical Center, Tokyo, Japan; 2 Nishikoyama Toya Medical Clinic-Diabetes and Cardiovascular Care, Tokyo, Japan

**Keywords:** Single-access strategy, Percutaneous coronary intervention, Mechanical circulatory support, IMPELLA CP, Case report

## Abstract

**Background:**

Complex cardiac arrest cases may require concurrent veno-arterial extracorporeal membrane oxygenation (VA-ECMO), left-ventricular unloading using Impella, and urgent percutaneous coronary intervention (PCI), vascular access sometimes becomes a procedural bottleneck. Conventional single-access Impella–PCI via a 14 Fr peel-away sheath expedites workflow but increases femoral bleeding risk; conversely, the lower-bleeding 16 Fr Medikit sheath used in Japan typically precludes true single-access PCI. We report a rescue strategy employing a coaxial 16 Fr/14 Fr peel-away/6 Fr sheath configuration to achieve single-access Impella-supported PCI when radial access was unobtainable.

**Case summary:**

A 53-year-old man in refractory ventricular fibrillation received VA-ECMO via right femoral cannulation; coronary angiography through the left femoral artery revealed subtotal proximal right-coronary-artery occlusion. Radial access was unobtainable. An Impella CP was implanted through a 14 Fr peel-away sheath coaxially inserted into a 16 Fr Medikit sheath placed in the left common femoral artery. A 6 Fr sheath was advanced through the same peel-away sheath, permitting single-access PCI and successful stent deployment. After revascularization the 6 Fr and 14 Fr sheaths were removed, leaving the Impella supported by the 16 Fr sheath without bleeding complications.

**Conclusion:**

A coaxial 16 Fr/14 Fr/6 Fr femoral strategy enables safe single-access Impella-supported PCI, combining procedural efficiency with a lower bleeding risk when radial routes are not feasible.

## Background

Cardiac arrest due to acute coronary occlusion often requires three simultaneous interventions: Veno-arterial extracorporeal membrane oxygenation (VA-ECMO) for perfusion, Impella-based left ventricular unloading, and urgent culprit-artery recanalization [1]. When profound shock or ongoing cardiopulmonary resuscitation precludes radial access, operators must choose between two femoral options: (1) dual puncture—safe but slow and bleeding-prone or (2) single-access via the 14 Fr peel-away sheath [2]—fast but carries a higher bleeding risk. In Japan, the 16 Fr Medikit sheath is preferred for Impella insertion because its intact haemostatic valve mitigates bleeding, yet its design blocks passage of a guiding catheter, again necessitating two punctures.

We describe a streamlined coaxial solution: a 14 Fr peel-away sheath and then a 6 Fr guiding-catheter sheath are inserted through the haemostatic 16 Fr Medikit sheath, allowing true single-access Impella-supported percutaneous coronary intervention (PCI) while preserving bleeding control. The present case illustrates the feasibility of this 16 Fr → 14 Fr → 6 Fr technique in a patient with refractory ventricular fibrillation who underwent VA-ECMO, left ventricular unloading, and successful PCI without vascular complications.

## Case report

**Fig. 1:**
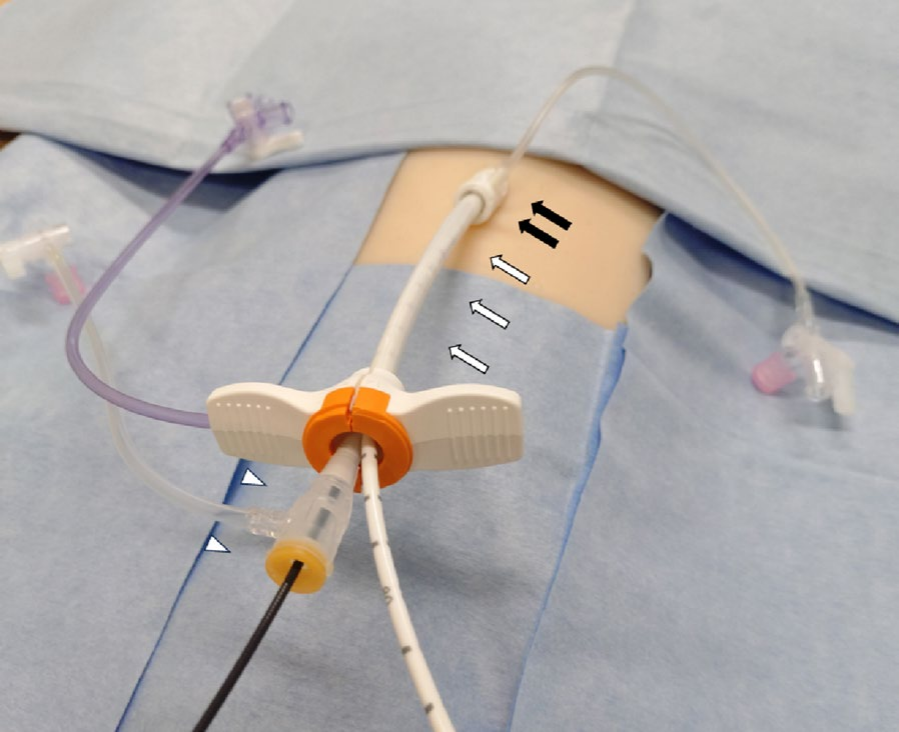
Single-access “sheath-in-sheath” setup. A 16 Fr Medikit femoral sheath (black arrow) contains a 14 Fr peel-away Impella introducer (white arrow) and an inner 6 Fr sheath (arrowhead) for the PCI guide, allowing Impella support and coronary intervention through one puncture

A 53-year-old man collapsed unexpectedly at work. Immediate bystander cardiopulmonary resuscitation was initiated, and emergency medical services arrived 9 min later to find the patient in ventricular fibrillation (VF). Four defibrillation attempts were unsuccessful in restoring spontaneous circulation. Thirty-six minutes after the initial collapse, the patient was transferred to our hospital in refractory VF. Given the prolonged cardiac arrest, extracorporeal cardiopulmonary resuscitation was commenced. VA-ECMO was established via right femoral cannulation. Radial access could not be secured in the arrest setting; therefore, diagnostic coronary angiography was performed through the left femoral artery and demonstrated a subtotal occlusion of the proximal right coronary artery (RCA). Prior to PCI, we opted for left ventricular unloading using an Impella CP device (Abiomed, Danvers, MA, USA). A 16 Fr sheath (Medikit, Tokyo, Japan) was placed in the left common femoral artery. Following Impella CP implantation, the patient spontaneously converted to sinus rhythm without electrical cardioversion. Through this, a 14 Fr peel-away sheath supplied with the Impella system was advanced, and the Impella catheter positioned across the aortic valve into the left ventricle. To achieve single-access PCI, a 6 Fr sheath was then introduced coaxially through the 14 Fr sheath, providing a conduit for the 6 Fr guiding catheter while the Impella remained in situ (Fig. 1). After the successful completion of PCI of the RCA, the 6 Fr sheath and, subsequently, the 14 Fr peel-away sheath were withdrawn, leaving the Impella supported by the 16 Fr sheath. To mitigate the risk of lower-extremity malperfusion associated with large-bore femoral access, antegrade perfusion sheaths were inserted into both femoral arteries, ensuring continuous distal limb perfusion throughout the subsequent mechanical support period.

## Discussion

Alternative single-access techniques using preclosure devices and puncturing the 14 Fr peel-away sheath have been reported by experienced operators; [3] however, in the Japanese regulatory context, concerns about bleeding from the peel-away sheath have prompted a preference for hemostatic large-bore sheaths, particularly in unstable arrest settings [4]. In Japan, the 16 Fr Medikit sheath is therefore preferred for Impella placement; however, its use traditionally precludes single-access PCI. The coaxial strategy outlined here combines the bleeding-risk advantage of the 16 Fr sheath with the procedural efficiency of single-access intervention when radial routes are unavailable. The use of a 7 Fr slender sheath is discouraged owing to tip fragility.

## Conclusion

Our case demonstrates that the 16 Fr → 14 Fr → 6 Fr configuration offers a practical balance between bleeding safety and interventional efficiency when radial access is impossible. Wider adoption may simplify complex cardiogenic-shock interventions and reduce vascular complications.

## Data Availability

No datasets were generated or analysed during the current study.

## Abbreviations

PCI: percutaneous coronary intervention
RCA: Right coronary artery
VA-ECMO: Veno-arterial extracorporeal membrane oxygenation
VF: Ventricular fibrillation
